# Immunogenicity and safety of the combined vaccine for measles, mumps, and rubella isolated or combined with the varicella component administered at 3-month intervals: randomised study

**DOI:** 10.1590/0074-02760180517

**Published:** 2019-03-07

**Authors:** Eliane Matos dos Santos, Tatiana Guimarães Noronha, Isabelle Soares Alves, Robson Leite de Souza Cruz, Clara Lucy de Vasconcellos Ferroco, Ricardo Cristiano Brum, Patricia Mouta Nunes de Oliveira, Marilda Mendonça Siqueira, Mariza Cristina Lima, Francisco Luzio de Paula Ramos, Camila de Marco Bragagnolo, Luiz Antonio Bastos Camacho, Maria de Lourdes de Sousa Maia

**Affiliations:** 1 Fundação Oswaldo Cruz-Fiocruz, Instituto de Tecnologia em Imunobiológicos, Bio-Manguinhos, Rio de Janeiro, RJ, Brasil; 2 Fundação Oswaldo Cruz-Fiocruz, Instituto Oswaldo Cruz, Laboratório de Vírus Respiratórios e Sarampo, Rio de Janeiro, RJ, Brasil; 3 Instituto Evandro Chagas, Belém, PA, Brasil; 4 Fundação Oswaldo Cruz-Fiocruz, Escola Nacional de Saúde Pública Sergio Arouca, Rio de Janeiro, RJ, Brasil

**Keywords:** vaccine, adverse events, measles, mumps, rubella, immunogenicity, safety, clinical trial

## Abstract

**BACKGROUND:**

Field testing required to license the combined measles, mumps, and rubella (MMR) vaccine must take into account the current recommendation of the vaccine in Brazil: first dose at 12 months and second dose at 15 months of age in combination with a varicella vaccine.

**OBJECTIVES:**

This study aimed to evaluate the clinical consistency, immunogenicity, and reactogenicity of three batches of MMR vaccine prepared with active pharmaceutical ingredients (API) from Bio-Manguinhos, Fiocruz (MMR-Bio), and compare it to a vaccine (MMR produced by GlaxoSmithKline) with different API.

**METHODS:**

This was a phase III, randomised, double-blind, non-inferiority study of the MMR-Bio administered in infants immunised at health care units in Pará, Brazil, from February 2015 to January 2016. Antibody levels were titrated by immunoenzymatic assays. Adverse events were recorded in diaries.

**FINDINGS:**

Seropositivity levels after MMR-Bio were 97.6% for measles, 84.7% for mumps, and 98.0% for rubella. After the MMRV vaccine, seroconversion rates and GMT increased substantially for mumps. In contrast, approximately 35% of the children had no detectable antibodies to varicella. Systemic adverse events were more frequent than local events.

**CONCLUSION:**

The demonstration of batch consistency and non-inferiority of the Bio-MMR vaccine completed the technology transfer. This is a significant technological achievement with implications for immunisation programs.

The combined vaccine for measles, mumps, and rubella (MMR) used since 2003 by the Brazilian National Immunisation Program (NIP) is a lyophilised mixed preparation of attenuated virus strains of measles (Schwarz strain), mumps (RIT 4385 strain ― derived from the Jeryl Lynn strain), and rubella (Wistar RA 27/3 strain).

The immunisation schedule of the MMR vaccine in Brazil was carried out with the first dose administered at the age of 12 months and the second dose at the age of four-six years. After 2013, the NIP introduced the measles, mumps, rubella (the same strains) and varicella vaccine [attenuated Oka strain (MMRV)] at the age of 15 months and administered the MMR at the age of 12 months. The administration of the MMRV vaccine at the age of 15 months would increase the coverage of the second dose of the MMR vaccine and the same time introduce the varicella vaccine, thereby eliminating an injection and a visit to health units.^1^

The use of two doses of MMR vaccine, or MMR vaccine followed by MMRV vaccine, increases the levels of antibody titres and enables high seroconversion for MMR, virtually eliminating primary failures and maintaining the levels of antibody titres for a prolonged period of time.^2^

There seems to be no advantage in delaying the administration of the second dose; that is, administering the dose when the child reaches the age four-six years.^3^

This study aimed to evaluate the reactogenicity and immunogenicity of the attenuated MMR vaccines produced with active pharmaceutical ingredients (API) from two producers, Bio-Manguinhos (MMR-Bio) and GlaxoSmithKline (GSK) (MMR-GSK), administered to children aged 12-19 months according to the vaccination plan adopted by the NIP. The MMRV vaccine was administered to all study participants three months after the MMR vaccine as in the immunisation schedule. During the literature search, none of the studies used the NIP’s recommended immunisation schedule for the MMR vaccine.

The MMR available in the public health network in Brazil is the result of the transfer of technology between the GSK laboratory and the Institute of Technology in Immunobiologicals [Bio-Manguinhos/Fiocruz (Bio-M)]. The primary objectives of the study were to demonstrate the clinical consistency of three consecutive batches of the MMR vaccine produced with API from Bio-Manguinhos (MMR1, MMR2, and MMR3) and the non-inferiority of this vaccine compared with that produced with API from GSK in terms of immunogenicity and reactogenicity. Although the reactogenicity and immunogenicity of the MMR vaccine are well known, this study complied with the requirement of the Brazilian National Regulatory Authority (ANVISA) for the licensing of a vaccine from a new API manufacturing site. This study also showed a better understanding of the performance of the MMR vaccine, with the immunisation schedule adopted by the NIP, using a sample of the target population for immunisation with the MMR vaccine followed by the MMRV vaccine.

## SUBJECTS AND METHODS

This was a phase III, randomised, double-blind, non-inferiority study conducted in three primary health care units and one school health centre in Belém, Pará, Brazil, from February 2015 to January 2016. The immune response and adverse events between the two groups of children immunised with one of the two combined measles-mumps-rubella vaccines were compared: MMR-Bio-M or MMR-GSK. After three months, both groups received the combined measles-mumps-rubella-varicella (MMRV) vaccine, as recommended by the Brazilian NIP. Their immune response was compared after the MMR vaccine and after the MMRV vaccine. Thus, the performance of the varicella component of the MMRV vaccine was a by-product of this study.

Participants were infants, aged 12-19 months and 29 days, healthy at the time of MMR vaccination, had no history of receiving live-attenuated vaccination in the last 30 days, without significant comorbidity, and who were brought to the health care units for routine vaccination. Infants were excluded from the research if they had received blood derivatives or blood transfusion, including immunoglobulins, in the previous 12 months, had a history of corticosteroid therapy in immunosuppressive doses or other immunosuppressants in the last six months, had a history of known systemic hypersensitivity to neomycin or any of the other components of the vaccine, or had severe allergy and anaphylaxis to egg proteins. Vaccination was postponed for children who had axillary temperature ≥ 37.5ºC on the day of vaccination or on the three previous days, and for children who took antibiotics seven days prior to the study. In these cases, the children were rescheduled for enrolment after 14 days of the disappearance of the fever and after discontinuing antibiotic treatment.

*Vaccines* - In order to demonstrate the consistency of production batches, three consecutive batches of the MMR vaccine with API were produced in MMR-Bio. The results of the three batches were pooled and compared with those of the vaccine with API from GSK. All the vaccines under study, regardless of the origin of the APIs, were produced by Bio-Manguinhos/Fiocruz (Rio de Janeiro/Brazil), under Good Manufacturing Practices. As for the MMRV vaccine, administered to all the research participants as the second dose of the MMR components, only one batch produced by GSK (TVV-GSK) was used.

The predicted interval between the MMR and MMRV vaccines was 90 days. The batches and potencies of the MMR-Bio vaccine were as follows: for MMR1-138VVA069Z, measles: 4.29 log_10_ CCID50/dose, mumps: 5.00 log_10_ CCID50/dose, and rubella: 3.86 log_10_ CCID50/dose: for MMR2-139VVA080Z, measles: 4.14 log_10_ CCID50/dose, mumps: 4.77 log_10_ CCID50/dose, and rubella: 4.00 log_10_ CCID50/dose; and for MMR3-139VVA081Z, measles: 3.91 log_10_ CCID50/dose, mumps: 4.61 log_10_ CCID50/dose, and rubella: 4.20 log_10_ CCID50/dose. The batch and potencies of the MMR-GSK vaccine were as follows: 13UVVA108Z, measles: 3.82 log_10_ CCID50/dose, mumps: 4.95 log_10_ CCID50/dose, and rubella: 3.80 log_10_ CCID50/dose. The batch and potencies of the MMRV-GSK vaccine were as follows: A71CA847A, measles: 3.77 log_10_ CCID50/dose, mumps: 4.87 log_10_ CCID50/dose, rubella: 3.73 log_10_ CCID50/dose, and varicella (Oka strain, attenuated): 4.3 log_10_ PFU/dose.

The MMR vaccine was placed in a multi-dose vial (10 doses/vial) and the MMRV vaccine was in a monodose syringe, with diluent, and lyophilised vial. However, to enable the randomisation proposed by the study, only one dose per vial with a number designated by prior draw was used for each participant. The vaccines were stored and administered according to the recommended dosage and route of administration indicated in the package insert. All vaccines were stored at + 2ºC to + 8ºC and were reconstituted immediately before administration. Participants received 0.5 mL of the MMR vaccine, as well as the MMRV vaccine, both administered subcutaneously in the deltoid region of the left arm. After administration of the vaccines, participants waited 30 min for observation, as a precaution for immediate adverse events. Other vaccines scheduled in the immunisation plan for specific age groups were administered in the usual vaccination room of the same primary health units and recorded in the data collection forms provide during the study.

*Randomisation and blinding* - All vials of the MMR vaccines were identical, labelled with numbers from 1 to 1,560 with random sequence of the types of vaccines. The randomisation list was drawn up by the BioForm system (electronic system used to record patients’ clinical information) with numbers from 1 to 1,560, which was not disclosed to the field work team in order to keep the blindness of the participants and the study team. Randomisation was conducted by blocks of size 4, with 390 infants in each arm; about 1,170 infants comprised the MMR-Bio group and 390 comprised the MMR-GSK group (3:1 allocation ratio).

At the first contact, after signing the consent form and blood collection, the child considered eligible for the study was randomised and assigned a participant identification code (PIC) generated by the BioForm system. The research centre team administered the vaccine from the vaccine vial with the corresponding PIC.

Only the statistician responsible for generating the random sequence, the supervisor of the Quality Assurance Department, and those responsible for the labelling of the Bio-Manguinhos vaccines had access to the list of randomised numbers. The randomisation list generated by BioForm was printed and sealed in envelopes deposited in the vault at Bio-Manguinhos. In case of need (e.g., occurrence of serious adverse events), the vaccine administered can be requested from the general coordinator of the study in Bio-Manguinhos to allow the clinical management of the case and to evaluate the implications for the continuity of the study.

*Sample size* - To calculate the sample size, the results of the immunogenicity used as reference were obtained in previous studies on combined MMR vaccines.^2^ ^,^ ^4^ ^)^ For a more conservative sample size calculation, the mean seropositivity for mumps was used as reference, as it was the antigen with the lowest immunogenicity. The current recommendations for sampling calculations and statistical analysis for non-inferiority or equivalence studies were also followed.^5^ ^,^ ^6^ Thus, a sample size was calculated to evaluate the consistency of the batches of the MMR-Bio vaccine with 80% power, a 90% confidence level, and a 10% equivalence margin. Moreover, a sample size was calculated to assess the non-inferiority of the MMR-Bio vaccine in relation to the MMR-GSK vaccine with 80% power, a 97.5% confidence level, 10% non-inferiority margin, and a 3:1 allocation ratio. The largest sample size (for batch consistency) was adopted, and 390 participants were allocated to each of the batches of the MMR-Bio vaccine and the MMR-GSK vaccine.

*Evaluation of the immunogenicity* - Blood samples were collected before each vaccine dose was administered and approximately 42-60 days after vaccination, considering the 51-day interval as ideal. Serum aliquots were sent to the National Reference Laboratory for Measles, Ministry of Health, Oswaldo Cruz Institute/Fiocruz for analysis. Antibody titres (immunoglobulin G ― IgG) against measles, mumps, rubella, and varicella were determined using Enzygnost (Siemens, Marburg, Germany) IgG enzyme-linked immunosorbent assay (ELISA) commercial kit. The cut-off points for seropositivity (antibody titres above the cut-off point) were ≥ 321 mIU/mL for measles, ≥ 457 U/mL for mumps, ≥ 10 IU/mL for rubella, and ≥ 101 mIU/mL for varicella. Samples with titres below these values were considered negative. All procedures were conducted in accordance with the kit instruction manual. Immunogenicity was defined by the seroconversion of seronegative infants before the vaccine test into seropositive infants after the vaccination test.

In addition, the plaque reduction neutralisation test (PRNT) was carried out for measles, using a titre of 1:25 as a cut-off point for seropositivity. The test was requested only if the ELISA showed seronegative results or the results were inconclusive for measles, for confirmation.

Batches were considered consistent when the lower and upper limits of the 95% confidence intervals of the difference in seropositivity between batch pairs were between −10% and +10%. Consistency was defined as follows: the 95% confidence interval of the geometric mean titre (GMT) ratio should be between 0.5 and 2.

Non-inferiority was defined as the difference in seropositivity rates for each of the antigens between the MMR-Bio vaccine and the MMR-GSK vaccine greater than or equal to −10% (closer to the null). More precisely, the null hypothesis that the seropositivity rate for MMR-Bio vaccines was inferior to that for MMR-GSK vaccines was rejected if the lower limit of the 95% confidence interval of the difference between the seropositivity rates of the vaccine under test and the vaccine of reference was above −10% (closer to the null), which indicated that the immunogenicity of the MMR vaccine under test was not inferior to the immunogenicity of the reference vaccine. Non-inferiority was defined as follows: the lower limit of the 95% confidence interval of the GMT ratio should be above 0.5.

*Safety and reactogenicity assessment* - The parents/guardians of the research participants were instructed to record local (pain, erythema, and oedema) and systemic adverse events (fever defined by axillary temperature ≥ 37.5ºC, exanthema, irritability, sleepiness, and loss of appetite) in the diary of adverse events for 10 days after vaccination (from D0 to D10). The intensity of symptoms such as pain, irritability, loss of appetite, and sleepiness was graded from 0 (absent) to 4 (very intense). The size of the oedema and erythema was measured using a ruler, and the largest diameter was recorded. The axillary temperature was checked using a mercury thermometer every morning after vaccination, and the guardian of the child was advised to administer an antipyretic medication in case of fever and verify the temperature several times during the day. Exanthema was graded from “absent” to “needing hospitalisation”.

Unsolicited adverse events were recorded up to 30 days after vaccination (from D0 to D30). Serious adverse events were recorded throughout the study. The causality of unsolicited adverse events and serious adverse events was assessed by the investigator in charge, the sub-investigators of the study, and the Independent Committee on Safety Monitoring.

*Statistical analysis* - To analyse the clinical consistency of the batches, the three consecutive production batches of the MMR-Bio vaccine were compared in terms of the proportions of seropositivity for each of the three antigens. Confidence intervals were constructed to determine the differences between these proportions by analysing the batches in pairs (MMR1-MMR2, MMR1-MMR3, and MMR2-MMR3), so that equivalence could be verified. Once batch equivalence was demonstrated, the non-inferiority analysis considered the immune response pooled for all three batches of the MMR-Bio vaccine compared with the MMR-GSK vaccine. The immunogenicity was analyse using “intention-to-treat” analysis, which covered all participants who were randomised, disregarding the violations of protocol, and “per protocol,” which included only those participants who had blood samples collected within the range provided in the protocol (42-60 days after vaccinations) and intervals of 42-90 days between vaccinations.

The rates of seroconversion, seropositivity, and GMTs of IgG, together with their 95% confidence intervals, were calculated for each component of the vaccines. The groups were compared using the chi-square test (seropositivity and seroconversion rates) and Mann-Whitney test (GMT) with a significance level of 0.05. In addition, the ratio between the GMTs and the difference in seropositivity and in seroconversion rates between the groups were calculated, as well as their 95% confidence intervals.

The reactogenicity all randomised participants was determined using “intention-to-treat” and “per protocol” analysis, which covered all participants who had filled the diary of adverse events and who completed the study. The groups were compared using the chi-square test with a significance level of 0.05. Data were analysed using the SPSS software, version 20.0 (SPSS, Inc., Chicago, Illinois).

*Ethical and regulatory approval* - The study was conducted in accordance with the Declaration of Helsinki, Resolution 466/2012 of the National Health Council/Ministry of Health, and the Guidelines for Good Clinical Practice. The study protocol was approved by the local Research Ethics Committees of the Evandro Chagas Institute (CAAE: 39416914.8.0000.0019) and the State University of Pará (CAAE: 20409813.9.3002.5170) and the ANVISA (file of the EC: 0009731154, in 07/Jan/2015). The study was recorded in the “ClinicalTrials.gov” database (registration no. NCT01991899). Informed consent was obtained from the parents or guardians of the participants before they were included in the study. The Independent Committee on Safety Monitoring, formed by vaccine experts, met during the study to assess the adverse events and scientific integrity of the study.

At the end of the study, after obtaining the serology results of the four antigens, a second dose of the MMRV vaccine was offered to the children who did not present seroconversion to one or more antigens.

## RESULTS

Altogether, 1,639 participants signed the informed consent, of which 1,563 were randomised and 1,560 received the MMR vaccines (392 with the API of GSK and 1,168 with the API of Bio-Manguinhos). Meanwhile, 1,472 received the MMRV vaccine, of which 36 received the routine MMRV vaccine from the health units (same vaccine under study, but from other batches).

After signing the informed consent, 79 of the 1,639 participants were excluded before receiving the MMR vaccine, mainly because of withdrawals and “other reasons”.

Of the 1,560 infants who received the MMR vaccine, 88 (5.6%) were excluded without receiving the MMRV vaccine and 17 (1.1%) were excluded after the MMRV vaccine, mainly because of “loss to follow-up” and “withdrawal”; a total of 105 (6.7%) participants were excluded (Figure).

**Figure:**
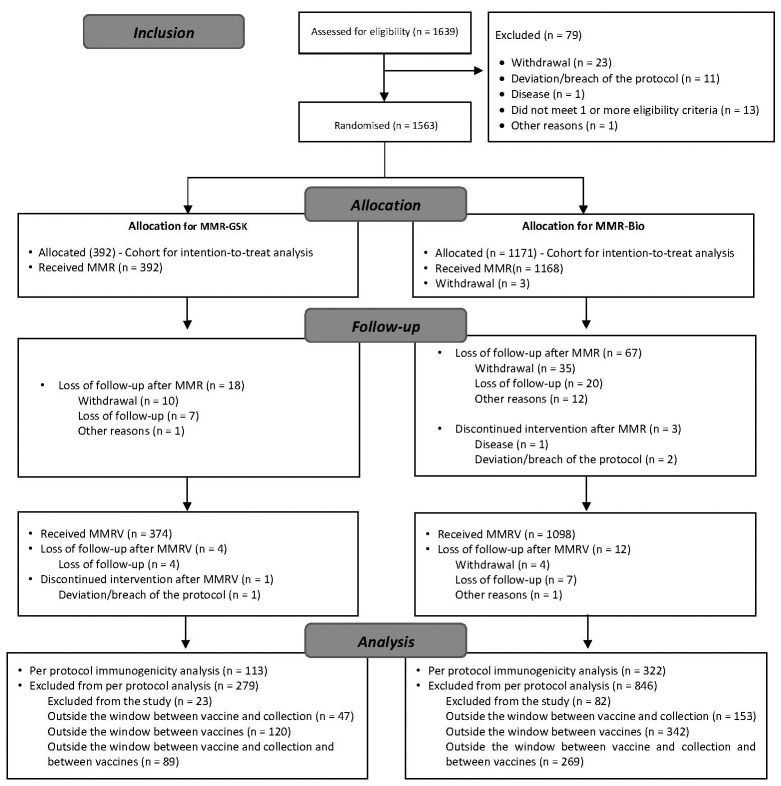
Flowchart of the participants in the various phases of the clinical trial.

The interval between the MMR vaccine and MMRV vaccines varied from 23 to 203 days (MMR-Bio and MMR-GSK both with a median of 91 days). The blood collection interval after the MMR vaccine ranged from 12 to 203 days (MMR-Bio and MMR-GSK, both with a median of 53 days), and the collection interval after the MMRV vaccine ranged from 35 to 181 days (MMR-Bio and MMR-GSK, both with a median of 51 days). A large proportion of participants fell outside the 42-60-day collection interval, which was one of the criteria to be included in the per protocol analysis.

The participants, on average, were at 12 months of age, with a slightly higher male proportion; they were eutrophic and there was a low proportion of seropositive infants prior to the vaccination test (Table I). The four groups (three batches of MMR-Bio vaccine and MMR-GSK vaccine) did not differ substantially in terms of baseline characteristics (data not shown).

**TABLE I:** Descriptive statistics of weight, age, and sex of the participants of the vaccine research

Variable	MMR-Bio (N = 1171)	MMR-GSK (N = 392)
Age (months)		
Minimum	12	12
Maximum	19	19
Median	12	12
Mean	13	13
SD	1,2	1,4
Sex		
Female (%)	47,6	48,2
Male (%)	52,4	51,8
Weight (g)		
Minimum	6180	6780
Maximum	14985	15500
Median	10000	9945
Mean	10104	10113
SD	1315	1321
Previously seropositive participants		
Measles (%)	2,2	2,7
Mumps (%)	1,6	2,7
Rubella (%)	1,2	2,7

GSK: GlaxoSmithKline; MMR: measles, mumps, and rubella; SD: standard deviation;

*Immunogenicity* - The evaluation of the clinical consistency of the three batches showed equivalence between them in the “per protocol” and “intention-to-treat” analyses. The differences in the seroconversion of antigens in the batch-to-batch comparison in the intention-to-treat analysis were as follows: batches 1 and 2, measles: -2.3% (-6.5; 1.9), mumps: -1.5% (-7.5; 4.4), and rubella” -1.0% (-5.1; 3.1); batches 1 and 3, measles: -1.8% (-5.9; 2.4), mumps: 4.2% (-1.6; 9.9), and rubella: 0.5% (-3.4; 4.5); and batches 2 and 3, measles: 0.5% (-3.8; 4.9), mumps: 5.7% (-0.1; 11.5), and rubella: 1.6% (-2.5; 5.6).The non-rejection of the difference in one batch did not affect the non-inferiority results of the TVV-Bio vaccine compared with the TVV-GSK vaccine (“data not shown”).

In the two comparison groups, the seropositivity levels after the MMR vaccine were more than 90%, except for the mumps component, in which 20% of the children were seronegative after the vaccine (Table II). The seroconversion rates were lower than the seropositivity for all components of the MMR-Bio and MMR-GSK. The geometric mean of the antibody titres of the two vaccines was similar, except for the measles component, in which the MMR-GSK vaccine had a 13% higher GMT than the MMR-Bio vaccine.

**TABLE II:** Results of seropositivity seroconversion and geometric mean titer (GMT) (intention-to-treat analyses)

Component	After MMR				After MMRV			
	MMR-Bio (N = 1171)		MMR-GSK (N = 392)		MMR-Bio (N = 1171)		MMR-GSK (N = 392)	
Measles								
Seropositivity (%) (CI 95%)	91,2	(89,6; 92,8)	93,6	(91,2; 96,1)	92,6	(91,1; 94,1)	94,1	(91,8; 96,5)
Difference of seropositivity (%) (CI 95%)	-2,4 (-5,6; 0,7)				-1,6 (-4,5; 1,4)			
GMT (CI 95%)	1929,8	(1703,3; 2186,5)	2439,4	(2008,7; 2962,5)	2118,0	(1861,8; 2409,4)	2487,5	(2025,4; 3054,9)
GMT ratio of MMR-Bio/MMR-GSK (CI 95%)	0,8 (0,6; 1)				0,9 (0,7; 1,1)			
Seroconversion (%) (CI 95%)	89,7	(87,9; 91,4)	91,8	(89,1; 94,6)	90,9	(89,3; 92,6)	92,3	(89,7; 95)
Difference of seroconversion (%) (CI 95%)	-2,2 (-5,6; 1,2)				-1,4 (-4,6; 1,8)			
Mumps								
Seropositivity (%) (CI 95%)	79,1	(76,7; 81,4)	80,6	(76,7; 84,5)	92,2	(90,7; 93,8)	93,6	(91,2; 96,1)
Difference of seropositivity (%) (CI 95%)	-1,5 (-6,2; 3,1)				-1,4 (-4,4; 1,6)			
GMT (CI 95%)	637,1	(569,5; 712,7)	730,6	(612,4; 871,6)	1898,3	(1672,1; 2155,1)	2351,5	(1917,4; 2883,9)
GMT ratio of MMR-Bio/MMR-GSK (CI 95%)	0,9 (0,7; 1,1)				0,8 (0,6; 1)			
Seroconversion (%) (CI 95%)	77,8	(75,4; 80,2)	79,1	(75; 83,1)	90,9	(89,2; 92,5)	92,1	(89,4; 94,8)
Difference of seroconversion (%) (CI 95%)	-1,3 (-6; 3,4)				-1,2 (-4,5; 2)			
Rubella								
Seropositivity (%) (CI 95%)	91,5	(89,9; 93,1)	92,9	(90,3; 95,4)	92,7	(91,3; 94,2)	94,1	(91,8; 96,5)
Difference of seropositivity (%) (CI 95%)	-1,3 (-4,4; 1,8)				-1,4 (-4,3; 1,5)			
GMT (CI 95%)	45,1	(42; 48,4)	50,1	(44,6; 56,3)	53,5	(49,8; 57,4)	59,6	(53; 67)
GMT ratio of MMR-Bio/MMR-GSK (CI 95%)	0,9 (0,8; 1)				0,9 (0,8; 1)			
Seroconversion (%) (CI 95%)	90,9	(89,2; 92,5)	91,1	(88,2; 93,9)	92,1	(90,5; 93,6)	92,3	(89,7; 95)
Difference of seroconversion (%) (CI 95%)	-0,2 (-3,5; 3,1)				-0,3 (-3,4; 2,8)			
Varicella								
Seropositivity (%) (CI 95%)	-	-	-	-	60,4	(57,6; 63,2)	59,9	(55,1; 64,8)
GMT (CI 95%)	-	-	-	-	83,4	(76,4; 91,1)	89,5	(77,3; 103,6)
GMT ratio of MMR-Bio/MMR-GSK (CI 95%)					0,9 (0,8; 1,1)			
Seroconversion (%) (CI 95%)	-	-	-	-	57,0	(54,1; 59,8)	54,6	(49,6; 59,5)

CI: confidence interval; GSK: GlaxoSmithKline; MMR: measles, mumps, and rubella; MMRV: measles, mumps, and rubella vaccine.

After the MMRV vaccine, the proportions of seropositivity, seroconversion, and the GMT increased substantially for the mumps component, and they slightly increased for the measles and rubella components (Table II). In contrast, approximately 40% of the children in both comparison groups had no immune response to the varicella component. These analyses were repeated and results were confirmed by the laboratory that performed the serological tests.

The proportions of seropositivity, seroconversion, and the GMT for all components were much higher in the per protocol analysis than in the intention-to-treat analysis (Table III). In the modified intention-to-treat analysis (analysis considering only participants in whom three serum samples were collected) (data not shown), the proportions of seropositivity, seroconversion, and the GMT were very similar to that of the per protocol analysis except for the varicella component, which showed a slightly lower GMT than in the per protocol analysis.

**TABLE III:** Results of seropositivity seroconversion and geometric mean titer (GMT) (per protocol analyses)

Component	After MMR				After MMRV			
	MMR-Bio (N = 322)		MMR-GSK (N = 113)		MMR-Bio (N = 322)		MMR-GSK (N = 113)	
Measles								
Seropositivity (%) (CI 95%)	97,2	(95,4; 99,0)	98,2	(95,8; 100,0)	100,0	-	100,0	-
Difference of seropositivity (%) (CI 95%)	-1,03 (-4,0; 2)				0,0			
GMT (CI 95%)	3069,0	(2791,9; 3373,6)	3457,3	(2962,7; 4034,5)	3783,8	(3510,1; 4078,8)	3885,7	(3420,2; 4414,6)
GMT ratio of MMR-Bio/MMR-GSK (CI 95%)	0,89 (0,7; 1,1)				1,0 (0,8; 1,1)			
Seroconversion (%) (CI 95%)	95,0	(92,6; 97,4)	95,6	(91,7; 99,4)	97,8	(96,2; 99,4)	97,3	(94,3; 100,0)
Difference of seroconversion (%) (CI 95%)	-0,5 (-5,0; 3,9)				0,5 (-2,9; 3,8)			
Mumps								
Seropositivity (%) (CI 95%)	86,0	(82,2; 89,8)	85,8	(79,3; 92,4)	99,4	(98,5; 100,0)	99,1	(97,4; 100,0)
Difference of seropositivity (%) (CI 95%)	0,2 (-7,3; 7,6)				0,3 (-1,7; 2,2)			
GMT (CI 95%)	1057,1	(950,2; 1176,0)	1054,9	(881,1; 1263,0)	3495,9	(3250,5; 3759,8)	4208,2	(3718,1; 4762,8)
GMT ratio of MMR-Bio/MMR-GSK (CI 95%)	1,0 (0,8; 1,2)				0,8 (0,7; 1,0)			
Seroconversion (%) (CI 95%)	84,5	(80,5; 88,4)	83,2	(76,2; 90,2)	97,8	(96,2; 99,4)	96,5	(93; 99,9)
Difference of seroconversion (%) (CI 95%)	1,3 (-6,7; 9,2)				1,4 (-2,4; 5,1)			
Rubella								
Seropositivity (%) (CI 95%)	97,5	(95,8; 99,2)	99,1	(97,4; 100,0)	100,0	-	100,0	-
Difference of seropositivity (%) (CI 95%)	-1,60 (-4,02; 0,82)				0,0			
GMT (CI 95%)	56,0	(51,5; 60,8)	61,4	(54,4; 69,3)	70,7	(66,3; 75,4)	77,8	(69,9; 86,5)
GMT ratio of MMR-Bio/MMR-GSK (CI 95%)	0,91 (0,8; 1,1)				0,9 (0,8; 1,0)			
Seroconversion (%) (CI 95%)	96,3	(94,2; 98,4)	96,5	(93,0; 99,9)	98,8	(97,5; 100,0)	97,3	(94,3; 100,0)
Difference of seroconversion (%) (CI 95%)	-0,2 (-4,2; 3,8)				1,4 (-1,8; 4,6)			
Varicella								
Seropositivity (%) (CI 95%)	-	-	-	-	71,4	(66,5; 76,4)	70,8	(62,3; 79,3)
GMT (CI 95%)	-	-	-	-	132,1	(119,6; 145,9)	143,8	(119,2; 173,4)
GMT ratio of MMR-Bio/MMR-GSK (CI 95%)					0,9 (0,8; 1,1)			
Seroconversion (%) (CI 95%)	-	-	-	-	68,3	(63,2; 73,4)	64,6	(55,6; 73,6)

CI: confidence interval; GMT: geometric mean titre; GSK: GlaxoSmithKline; MMR: measles, mumps, and rubella; MMRV: measles, mumps, and rubella vaccine.

The neutralisation test (PRNT) for measles was performed on serum samples of two participants who had an inconclusive and negative ELISA after the MMRV vaccine. The results of the PRNT showed that the inconclusive ELISA sample were weakly positive for antibody titres against measles, whilst the negative ELISA sample were negative for antibody titres against measles.

*Safety and reactogenicity* - When the three batches of the MMR-Bio vaccine were compared pairwise, batch 1 had a higher incidence of oedema (5.6%, batch 1 and 2.5%, batch 2) and pain (29.4%, batch 1 and 22.2%, batch 2) at the site of the vaccine, with mild intensity, than batches 2 and 3, respectively, with a significant difference. However, considering the total frequencies of solicited and unsolicited adverse events among the vaccine batches, no significant difference was observed.

Systemic adverse events (69.0%, MMR-Bio; 67.8%, MMR-GSK) more frequently occurred than local events (29.7%, MMR-Bio; 31.2%, MMR-GSK). Of the systemic events, the most frequent were sleepiness, loss of appetite, and fever, whilst the most frequent local adverse event was pain at the site of injection followed by erythema (redness) (Table IV). Adverse events were predominantly mild, and the frequency did not differ significantly in the comparison groups.

**TABLE IV:** Frequency of adverse events per vaccine occurring within 10 days after the measles, mumps, and rubella (MMR) vaccine

Adverse event	MMR-Bio		MMR-GSK		p-value
	Nº^*^	(%)	Nº^*^	(%)	
Local					
Pain	279	25,7	104	28,2	0,347
Edema	42	3,9	10	2,7	0,301
Redness	50	4,6	10	2,7	0,114
Secretion	11	1,0	4	1,1	1,00*
Systemic					
Irritability	325	29,9	101	27,4	0,351
Loss of appetite	477	43,9	157	42,5	0,907
Red spots on the skin	107	9,9	30	8,1	0,645
Sleepiness	498	45,9	162	43,9	0,328
Fever	307	28,3	100	27,1	0,666

GSK: GlaxoSmithKline; *: number of participants.

Axillary temperatures between 37.5ºC and 38.4ºC were most frequently observed among participants who presented with fever, mainly from D7 to D10: D7 (7.3%, MMR-Bio and 4.1%, MMR-GSK), D8 (8.3%, MMR-Bio and 5.7%, MMR-GSK), D9 (4.5%, MMR-Bio and 5.7%, MMR-GSK), and D10 (3.9%, MMR-Bio and 4.9%, MMR-GSK). The difference in the frequency of fever on days D4 (5.8%, MMR-Bio and 1.6%, MMR-GSK), D7 (12.1%, MMR-Bio and 6.8%, MMR-GSK), and D8 (14.9%, MMR-Bio and 9.5%, MMR-GSK) between the two groups was considered significant. The frequency of temperatures higher than 39ºC within 10 days of vaccination was less than 1%.

Of the unsolicited adverse events (reported from D0 to D10 in the descriptive field of the diary of adverse events or recorded from D11 to D30 in the data collection form of the study to record unsolicited adverse events), the most frequent adverse events were fever (15.3%, MMR-Bio and 14.4%, MMR-GSK) and diarrhoea (15.1%, MMR-Bio and 14.9%, MMR-GSK), without a significant difference between vaccines.

Of the 28 severe adverse events reported (21, MMR-Bio; 7, MMR-GSK), the most frequent were asthma and gastroenteritis. All were cured without sequelae, and none had the confirmed causality with the vaccine.

Among the drugs used during the study, the most frequent were antipyretics (20.1%, MMR-Bio; 20%, MMR-GSK), followed by antibiotics (15.4%, MMR-Bio; 14%, MMR-GSK), and bronchodilators (10.2%, MMR-Bio; 9.4%, MMR-GSK).

## DISCUSSION

The study demonstrated the high immunogenicity of the measles and rubella components and the modest immunogenicity of the mumps component of the MMR vaccine with the active pharmaceutical ingredient from Bio-Manguinhos, which proved non-inferiority to the vaccine with API from GSK. Reactogenicity was also very low for both vaccines. This field assay with the vaccine provides valuable information for the NIP by showing the levels of safety and immunogenicity in a controlled study. However, the scientific rigor was reconciled with “real-life” conditions of primary health units in the less prosperous region of the country, with greater challenges and more limitations to the actions of immunisation.

The results also disclosed the poor immune response of the varicella component, which was unexpected. The MMRV vaccine at 15 months was an imposition of the basic immunisation schedule in Brazil. It allowed the study to show that the immune status of children who completed the immunisation schedule was satisfactory for measles, mumps and rubella, but insufficient for varicella.

A low seropositivity rate (< 3%) was observed in the initial visit prior to the administration of MMR vaccine (at least one of the three components of the vaccine) in the two comparison groups. This finding suggests the persistence of circulating maternal antibodies at 12 months of age.^7^ However, other explanations are plausible. The probability of false-positive results is small considering the high specificity of the tests used. Previous natural infection was also unlikely in those infants as the number of cases of the target diseases reported in that area within the period of the study was negligible (one case of congenital rubella syndrome in 2015).

The seroconversion rates for measles and rubella following the MMR vaccine were similar to those reported in previous studies.^2^ ^,^ ^8^ ^,^ ^9^ ^,^ ^10^ ^,^ ^11^ ^,^ ^12^ ^,^ ^13^ ^,^ ^14^ ^,^ ^15^ ^,^ ^16^ A smaller seroconversion for mumps was expected, in relation to measles and rubella, according to similar findings from other studies.^2^ ^,^ ^4^ ^,^ ^9^ ^,^ ^11^ ^,^ ^12^ ^,^ ^13^ ^,^ ^14^ ^,^ ^15^ Notable, mumps component is less immunogenic than the other two components of the MMR vaccine.

After two doses of the vaccines with MMR components, seroconversion was greater than 99% for all three components, using the same ELISA methodology for immunogenicity analysis.^14^ ^,^ ^17^ ^,^ ^18^ ^,^ ^19^

The geometric mean titres of MMR were similar to those of previous studies that used the same methodology to evaluate immunogenicity.^2^ ^,^ ^4^ ^,^ ^9^ ^,^ ^10^ ^,^ ^11^ ^,^ ^13^ ^,^ ^14^ ^,^ ^15^ ^,^ ^16^ ^,^ ^17^ ^,^ ^19^ ^,^ ^20^ The magnitude of the increase in the proportion of seropositives and GMT of antibodies for mumps after the second dose was much higher than that for measles and rubella, which is consistent with that observed in previous studies.^2^ ^,^ ^20^ and reinforces the need for a second dose of the mumps vaccine. In fact, as of 2006, the Advisory Committee on Immunization Practices (ACIP) of the United States formally recommended the administration of the second dose of mumps vaccine.^21^ ^)^ For the three MMR vaccine components, the booster dose possibly prolonged the protection against the target diseases.

The seroconversion rates (ELISA, Enzygnost Anti-VZV/IgG) of the varicella component of MMRV in this study ― 64.3% in the MMR-GSK group and 68.6% in the MMR-Bio group, with similar GMTs ― were substantially lower than those reported in previous studies.^18^ ^,^ ^22^ ^,^ ^23^ In the present study, IgG was titrated with three different lots of Enzygnost Anti-VZV/IgG. Unbeknownst to the laboratory personnel, one of them had a probable impaired accuracy according to a report from the manufacturer. However, the seropositivity post-MMRV immunisation for the sera tested with the suspect inaccurate lot (n = 332) was 59 (6%) and that for the sera tested with the other two lots (n = 1123) was 66, 3%.

Although the ELISA IgG is not considered sensitive enough to detect seroconversion after immunisation since, it is calibrated for the diagnosis of natural infection.^24^ The seropositivity was too low to be justified solely by test accuracy. In the work of Huang LM et al.,^18^ the GMT of varicella, after the second dose of the MMRV vaccine, increased approximately 20 times, suggesting that the first dose failed to seroconvert a number of vaccinees. In fact, the manufacturer of the MMRV vaccine recommended the administration of two doses with an interval of at least 4 weeks (insert package of the MMRV vaccine, GSK). Although the manufacturer of the MMRV vaccine and the ACIP^21^ recommended two doses of the varicella vaccine, the levels of seroconversion for the varicella component revealed in this study, if confirmed in other studies, do not seem to be acceptable.

Live-attenuated vaccines are prone to variations in potency across production lots, which is inherent to the manufacturing process. For measles, the largest difference between the lots (0.38 log10) meant that the potency of one batch was 2.4 times that of the other. Similarly, the ratio of potency of batches of mumps and rubella components were 2.4 and 2.2, respectively. The study showed that the variation, which is likely to occur in the regular supply of vaccines to the NIP, did not have a relevant impact on immunogenicity (as a proxy for protection).

The reactogenicity profile of the MMR vaccine of the two groups was similar for most of the solicited and unsolicited adverse events as described in previous studies.^8^ ^,^ ^11^ ^,^ ^13^ ^,^ ^15^ Systemic signs and symptoms are non-specific and do not differ from coincident health problems in children. Significant differences in the frequencies of pain and oedema for batch 1, compared with batches 2 and 3 of the MMR-Bio vaccine, led to the review of the quality of the batches, and no evidence was found to justify these differences.

Both vaccines were well tolerated by the participants, and no serious adverse events had confirmed causality for the vaccine. The frequency of fever was less than to that described in the Adverse Event Handbook of the Ministry of Health.^25^

The main virtue of this work, the experimental design with randomisation and strict control of the conditions of intervention application and measurement of the outcomes of interest, may also be its main limitation insofar as the performance of the vaccines may not be as good in the typical conditions of the units of the public health network. Immunogenicity may be lower in routine vaccination because of nonconformities in the conservation and application of vaccines, and reactogenicity may be greater because some of the exclusion criteria in this study were not applied to routine vaccination. Another aspect to be considered is that immunogenicity is an approximation of the vaccine efficacy, although the experience with these vaccines gives credence to seropositivity as a surrogate marker of actual protection. Additionally, a large proportion of participants were not included in the per protocol analysis mainly because of large blood collection intervals. However, this did not appear to have affected the results, which were very similar to those of the intention-to-treat analysis.

In conclusion, the results of this study showed that the MMR vaccine with API from Bio-Manguinhos, to be used in the public service network of Brazil, showed similar safety, reactogenicity, and immunogenicity profiles as those with original API in infants aged 12-19 months.

The immunisation plan of the NIP appears to be safe and immunogenic for the three components of the MMR, and immunity after two doses is long lasting. For varicella, the administration of a second dose should also be evaluated considering the performance of the vaccine and the epidemiological data of the disease in the country.

We considered that the human and material resources used, as well as the time spent by the participants, were justified as they allowed the demonstration of the immunogenicity and safety of a vaccine in the Brazilian immunisation program. The clinical validation of the batches of vaccines produced in Brazil completed the process of mastering the technology of production of a strategic material for the national immunisation program. The national production of this vaccine represents resource savings and reduces the vulnerability of the NIP to the interruption of supply by foreign laboratories in cases where vaccine shortage occurs in the world market. The poor performance of the varicella component of the MMRV vaccine needs to be better assessed given its implications in the National Immunisation Programs of Brazil and other countries that use or will introduce one or two doses of the varicella component. In 2018, the Brazilian Ministry of Health implemented the administration of the second dose of the varicella vaccine to children aged four-six years.^26^
